# Influence of Opponent Knowledge and Competitive Level on the Cognitive Processes of Decision-Making in High School Kendo Athletes: An Exploratory Study

**DOI:** 10.3390/sports14080320

**Published:** 2026-08-01

**Authors:** Yoichiro Sato, Masaki Eiga, Kenshiro Matsuzaki

**Affiliations:** 1 Faculty of Health Sciences, Hokkaido University of Science, Sapporo 0068585, Hokkaido, Japan; 2 Tokai University Sapporo Senior High School, Sapporo 0050825, Hokkaido, Japan; masa-21.kendo@outlook.jp; 3 Institute of Health and Sport Sciences, University of Tsukuba, Tsukuba 3058577, Ibaraki, Japan; matsuzaki.kenshir.kb@u.tsukuba.ac.jp

**Keywords:** cue utilization, prior information, cognitive mechanisms, elite athletes, action anticipation

## Abstract

In competitive sports, players must make decisions rapidly and accurately. However, the cognitive process underlying decision-making while integrating opponent knowledge and relative competitive level remains unclear. This study explored whether these conditions influenced the cognitive process underlying decision-making in kendo athletes. Thirty high-school kendo experts (higher- and lower-skilled groups) observed temporally occluded videos of “kote” (wrist) and “men” (head) actions performed by a familiar high-level model and an unfamiliar intermediate-level model. They anticipated the strike and reported the reason for their anticipation (“intuition,” “sword tip,” or “whole body”) or answered “uncertain.” A fisher’s exact test was performed for statistical analysis. “Uncertain” responses were significantly higher for the intermediate-level model during non-kinematic information stages, suggesting that knowledge about the opponent supported decision-making as non-kinematic information. Furthermore, accuracy rates were also significantly higher for the intermediate-level model immediately before the strike. This finding suggested that the difference in competitive level between observers and models influenced the cognitive processes involved in the tentative stage of decision-making. This study provides novel perspectives on the decision-making paradigm by suggesting that experts’ cognitive mechanisms differ according to their opponent knowledge and relative competitive levels.

## 1. Introduction

Success in highly competitive sports such as kendo depends on the ability for rapid decision-making. This ability is crucial for adapting to rapidly changing uncertain situations, as it helps athletes plan actions in advance rather than merely react. Therefore, for effective decision-making, a central role is played not by the execution of movement itself, but by action anticipation, which allows athletes to plan their responses to an opponent’s action as early as possible.

To date, action anticipation research has focused predominantly on ball games [1,2,3,4,5,6,7,8]. Previous research has consistently demonstrated that the accuracy of action anticipation varies substantially depending on an individual’s level of expertise. Generally, compared with novices, expert athletes can anticipate the outcome of others’ actions faster and more accurately [1,2,3,4,5]. This advantage stems from their specialized ability to perceive subtle kinematic features of the observed actions [1,5,6,7,8,9]. In addition to kinematic cues, non-kinematic information about the opponent, such as contextual priors (i.e., situational information including the opponent’s behavior patterns, preferences, and court position), plays a crucial role in anticipation [8,10,11]. This information provides an advantage to experts in inferring the opponent’s intentions at an early stage [8].

In recent years, reports have emerged regarding decision-making and action anticipation in combat sports [12,13,14,15]. Combat sports place athletes under high cognitive load because offense and defense are often required simultaneously [16]. Furthermore, because a single decision-making error can lead to immediate defeat, highlighting the critical importance of the speed and accuracy of anticipation. According to a systematic review of combat sports, expert athletes are more rapid and accurate than novices, particularly when facing an opponent’s attack [14]. Conversely, findings from studies comparing different levels of expertise (e.g., high- vs. low-level athletes) are inconsistent. A previous report has indicated that high-level athletes possess higher accuracy [14]. While others, as reported on fencing, suggest no significant difference in the anticipation accuracy on the basis of physical fitness levels among elite athletes [12]. Given that kendo involves the use of a weapon (sword), the findings may be similar to those in fencing; however, no studies have compared anticipation across different competitive levels in kendo. Furthermore, while anticipation accuracy likely varies with both the observer’s skill level and the relative difference in expertise between the observer and the model, no research has yet incorporated this specific condition.

Similar to ball games, non-kinematic information may influence decision-making in combat sports. In combat sports, this primarily refers to knowledge of the opponent’s movement patterns (habits) rather than court positioning due to the nature of close-range interactions. This knowledge of the opponent potentially serves as critical information for decision-making before kinematic information even appears; however, its specific role in combat sports remains unclear. Additionally, specific factors and philosophies unique to combat sports are reported to influence the cognitive processes involved in decision-making. In judo, rapid weight loss prior to competition has been shown to have adverse effects on cognitive performance [15]. In kendo, a unique perceptual concept known as “Enzan no Metsuke” (gazing at the far mountain) has been reported [13]. This report suggests that focusing one’s vision on the opponent’s head allows the observer to perceive the whole body. We believe it is necessary to use verbal reports to identify cognitive factors that influence decision-making, elements that cannot be captured by eye-tracking alone.

To clarify these unresolved points, this study focused on the cognitive process of decision-making in kendo to investigate whether conditions integrating knowledge of the opponent and competitive level (including the relative difference in skill level between the observer and the model) influence decision-making. By elucidating these points, this study may advance our understanding of the cognitive processes underlying superior decision-making ability in high-level kendo athletes and provide a new perspective on the decision-making paradigm.

## 2. Materials and Methods

### 2.1. Participants

Thirty high-school kendo experts with normal or corrected-to-normal vision participated in this study. The sample size was determined with reference to similar previous studies [1,17,18,19]. They were included in the sample because their stance was chūdan (center-of-body stance). Participants using jōdan were excluded because the lead foot differs from that in chūdan, which may affect the viewing perspective. Moreover, this stance was not included because of its limited competitive population. They were classified into higher- and lower-skilled groups based on two kendo coaches, who engaged in daily training sessions with all the participants and reached a consensus on group assignments based on the participants’ history of match performance, including the match results. The two expert kendo coaches were selected based on their dan (both had fifth dan rank) and years (10) of coaching experience. The higher-skilled group comprised participants competing at levels ranging from the high prefecture level to the national level, whereas the lower-skilled group comprised those competing from the local prefecture level to the prefecture (Hokkaido) levels. Detailed information on both groups is presented in Table 1. Participants were categorized according to their expertise level in competition rather than their Dan grade; thus, the Dan grade did not always correspond to actual match performance. As a result, 2nd and 3rd Dan athletes were included in both the higher- and lower-skilled groups.

The groups did not differ significantly in terms of basic physical characteristics or attained rank (dan level). All participants provided written informed consent before participating in the study. Additionally, for the minor participants, their parents provided written informed consent to the authors. This study was approved by the Research Ethics Committee of Hokkaido University of Science (approval no. 749) and complied with the ethical standards of the Declaration of Helsinki.

### 2.2. Stimulus Materials

**Figure 1:**
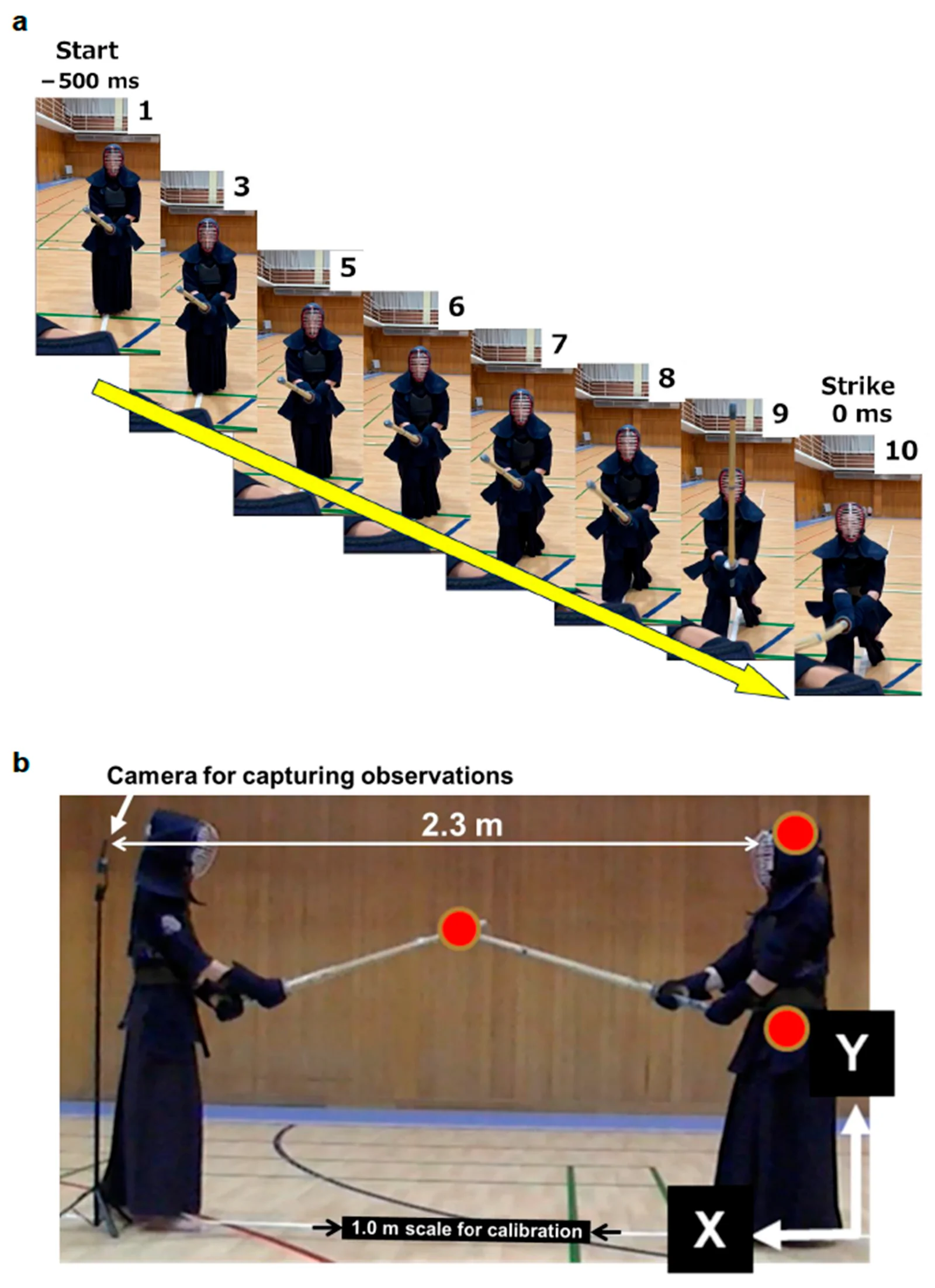
Model action examples. (**a**): Typical images of model action from clip start (−500 ms) to end (0 ms). (**b**): Side view of the model action. Notes: The red circles show landmarks for motion analysis (sword, head, and pelvis).

Video clips capturing kendo-striking movements by two experienced kendo practitioners were used as the visual stimuli for the action anticipation task. A high-ranked practitioner (kendo fifth dan; 27 years old; high-level model) and a middle-ranked practitioner (kendo second dan; 14 years old; intermediate-level model) performed the movements. Written informed consent to participate as a model in this study was provided by both models. Additionally, for the minor model, his parent provided written informed consent. The observers had lower competitive levels than the high-level model and higher competitive levels than the intermediate-level model. The high-level model was the participants’ coach, who regularly practiced with them; therefore, the observers were familiar with the opponent. However, the participants saw the intermediate-level player for the first time; thus, they did not have prior knowledge of this opponent. These two models were selected because the integration of the model’s expertise level and the observer’s familiarity with them allows for the following distinctions. If correct responses increase at an earlier stage for the intermediate-level model than for the higher-level model, it would indicate that the model’s expertise level is the primary factor influencing the results; this is because, all else being equal, knowledge of the opponent should typically lead to earlier anticipation compared to observing an unfamiliar individual. Conversely, if the observation of the intermediate-level model results in a higher frequency of ‘uncertain’ responses (i.e., ‘I don’t know’) than that of the higher-level model, it would suggest that knowledge of the opponent (acting as a contextual prior) is the dominant influence. This interpretation is based on the premise that lower-skilled models typically provide more obvious kinematic cues, which should theoretically lead to fewer uncertain responses unless the lack of familiarity significantly impairs the observer’s decision-making process. Both models performed “kote” (wrist) and “men” (head) strikes against the “motodachi” (opponent) from the “issoku ittō no maai” (one-step, one-sword interval) and from the same starting position. Both models were instructed to perform movements in a manner that made it difficult to distinguish between the two strikes. This action was recorded using a smartphone (iPhone 12 Pro, Apple Co., Cupertino, CA, USA, 60 fps) placed directly in front of the model (i.e., just above the motodachi’s right shoulder; Figure 1a) for observation purposes. Simultaneously, the models’ movements were recorded from the left side using a high-speed camera (GC-LJ20B, Q’sfix, Tokyo, Japan, 240 fps; Figure 1b) for motion analysis. The distance between the camera and the model’s head was 2.3 m. In kendo, the striking action is fundamentally executed by swinging with the left hand regardless of the practitioner’s dominant arm, while the right hand merely provides support. Consequently, the actions were recorded from a left-side perspective.

The videos recorded from the front of both models were edited to form 10 clips of different lengths, starting 500 milliseconds before the moment of impact (the striking moment) and creating temporally occluded clips (Figure 1a). Wondershare Filmora 13 (Wondershare Technology Inc., Hong Kong, China) was used to edit the clips for temporal occlusion. The duration of each video clip is shown in Table 2. Since the movement accelerated immediately before the strike, the intervals for segmenting the latter half of the video were shorter than those for the first half. A total of 40 videos were produced for the database, representing different combinations of the models (high- and intermediate-level), type of strike (kote and men), and clip length. The observation videos were created quasi-randomly by partially counterbalancing the combination of the models, type of strike, and clip length, ensuring that the high- and intermediate-level models, kote and men strikes, and 10 clip lengths appeared in appropriate proportions.

### 2.3. Procedure

The participants were asked to observe videos of the models’ movements, which varied in length, and to predict the strike outcome. Each video clip was displayed on a laptop (Lenovo YOGA, 14-inch, Beijing, China). The participants sat facing the laptop and practiced the answering method and computer operation by observing two practice videos before the main session. The entire session, including the explanation of the experimental procedure, lasted approximately 15 min. The observations were conducted in a separate space specifically set aside for this purpose within the club activity area. Although some background noise from the practice sessions was audible, the testing was performed in a generally quiet environment. They started the practice and main session videos by pressing a button on the laptop. Each video clip disappeared immediately after completion. Referring to Aglioti [1], three choices were provided: “kote,” “men,” and “I don’t know (uncertain).” The participants answered by circling the corresponding items on paper. For videos answered as “kote” or “men,” they were asked to verbally state the reason for their prediction. For example, they could state that the tip of the sword seemed to rise. Answers such as “just a feeling” or “intuition” were also acceptable. Verbal responses were recorded with a digital high-definition camera (HC-V495M, Panasonic, Osaka, Japan) for review. After observing one video, the participant could operate the laptop to start observing the next video. Each participant viewed 20 quasi-random videos selected from the database, consisting of selected combinations of model, strike type, and clip length.

### 2.4. Data Analysis

The numbers of correct, incorrect, and “uncertain” answers were counted for each clip. The proportions of each response type were also calculated. Reasons for answering “kote” or “men” were categorized into three groups (Table 3): “intuition,” “sword tip,” and “whole body.” “Intuition” included responses such as “just a feeling” or “hunch”; “sword tip” included responses such as “the tip of the bamboo sword went up/down” or “hands went up”; and “whole body” included responses such as “the upper body lowered” or “body movement was large.” The coding was performed by one of the authors, who was blinded to both the group assignments and the model conditions. This categorization helped clarify whether differences in observation focus could be detected even with subtle differences in skill level, with reference to Meyer’s [20] finding that experts use whole-body physical information more than novices do to predict actions. Furthermore, while previous studies examining cognitive mechanisms have used eye tracking methods [5,20], the duration of gaze fixation may not indicate which information participants used for prediction. Additionally, because the final judgment might be “intuition” even if bodily information was considered, verbal responses, including “intuition,” were included in this study.

To verify the movement kinematics of the models in the observed videos, footage recorded simultaneously from a side-on perspective using the digital high-definition camera was used for motion analysis. The analysis was conducted using Frame-Dias VI software (Q’sfix, Tokyo, Japan). The coordinates in the sagittal plane were identified for the head (vertex), pelvis (iliac crest level), and sword tip (bamboo sword tip). Prior to the recording of movements, calibration was established using a 1 m tape adhered to the floor along the line of motion, with a 1 m stand placed directly upon it. While the digitization process primarily employed automatic tracking functions, manual digitization was required for specific segments involving high-speed movements. All manual digitization was conducted by a single investigator.

The anteroposterior and vertical velocities were calculated using these positional coordinates. The time period for identifying the coordinates was 500 milliseconds, spanning 500 milliseconds before the moment of impact (t = 0 s), similar to that of the observation videos. Furthermore, the average velocity of each body part was calculated for each of the 10 observed movement segments to examine movement differences between the models. For example, for the first segment, the average velocity was calculated between −500 and −350 milliseconds and, for the second, between −350 and −300 milliseconds.

### 2.5. Statistical Analysis

A chi-squared test or Fisher’s exact test (performed when more than 20% of the cells had expected counts less than 5) was used for the following analyses:

1. Comparison of the proportions of correct, incorrect, and “uncertain” answers between the higher- and lower-skilled groups for each clip.
2. Comparison of the proportions of correct, incorrect, and “uncertain” answers for all participants between the high- and intermediate-level models for each clip.
3. Comparison of the proportions of reported reasons for selecting kote or men between the higher- and lower-skilled groups.

The Benjamini–Hochberg (BH) procedure was applied to adjust for multiple comparisons. Inter-rater reliability for the categorization of reasons provided in the verbal reports among the three raters was assessed using Fleiss’ kappa coefficient. The kappa values were interpreted according to the criteria proposed by Landis and Koch [21]. The overall kappa coefficient across the three categories was 0.981 (95% confidence interval [CI]: 0.902–1.06, *p* < 0.001). Specifically, the kappa values for each category were 1.000 for “Intuition” (95% CI: 0.891–1.109, *p* < 0.001), 0.973 for “Sword tip” (95% CI: 0.864–1.082, *p* < 0.001), and 0.963 for “Whole body” (95% CI: 0.854–1.072, *p* < 0.001). Any discrepancies in the coding were resolved through discussion among the authors to reach a final classification.

To assess intra-rater reliability for the segments of the motion analysis requiring manual digitization, the intraclass correlation coefficient (ICC) was calculated. For this reliability assessment, 24 data points from a high-motion segment (representing a 0.1 s interval) were selected and digitized five times. ICC (1,5) values and their 95% CIs were determined for both the X-coordinates (antero-posterior direction) and Y-coordinates (vertical direction). The ICC (1,5) for the X-axis was 0.996 (95% CI: 0.992–0.998), and for the Y-axis, it was 0.999 (95% CI: 0.998–1.000). SPSS version 31 (IBM Co., Armonk, NY, USA) and EZR version 1.70 (Jichi Medical University, Tochigi, Japan), which is a graphical user interface for R 4.5.2 (The R Foundation for Statistical Computing, Vienna, Austria) were used for the statistical analysis. EZR was employed specifically for Fisher’s exact tests. For all analyses, the significance level was set at *p* < 0.05.

## 3. Results

After adjusting for multiple comparisons using the BH method, none of the chi-squared or Fisher’s exact tests reached significance. Therefore, the following results are presented using unadjusted *p*-values and effect sizes.

**Figure 2:**
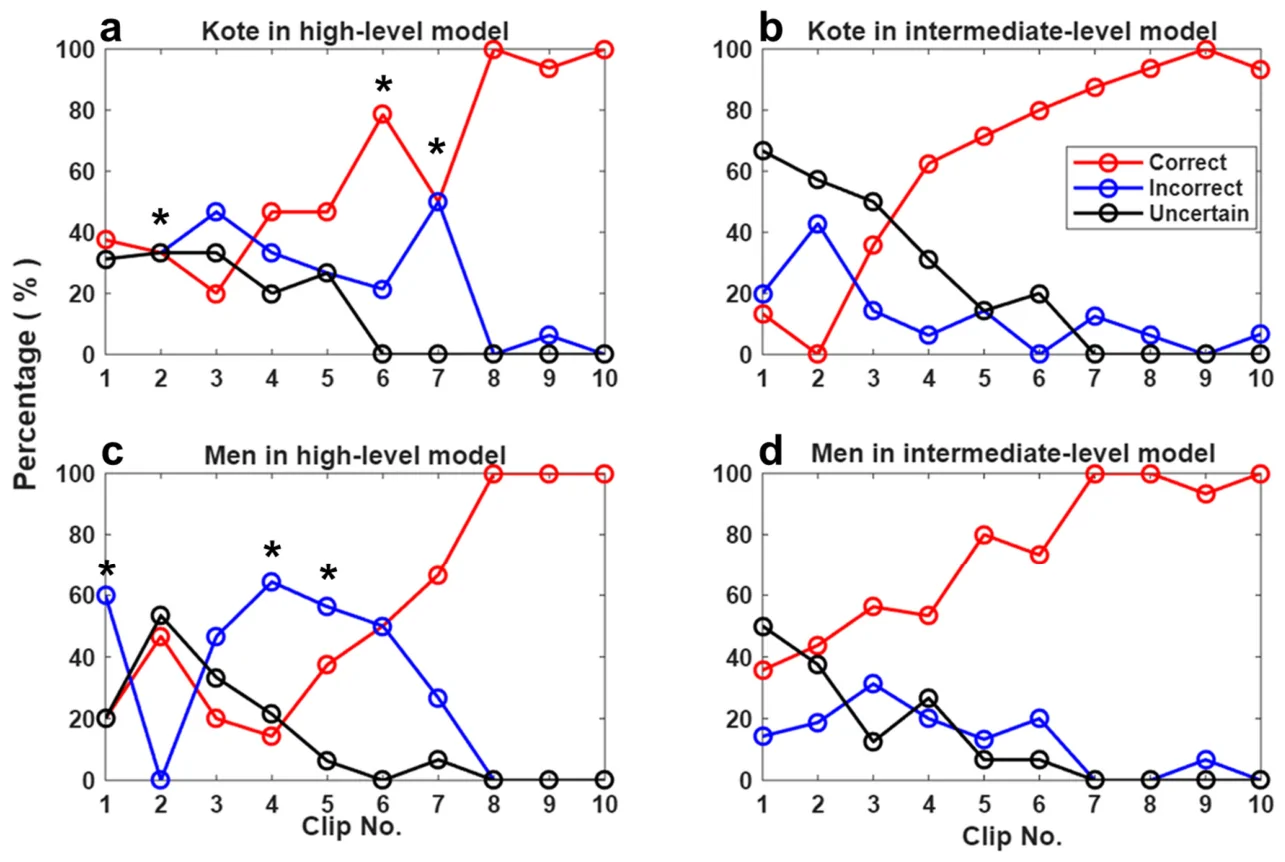
Response rates for both models’ kote and men clips for all participants. Notes. *: Significant difference between the high- and intermediate-level models.

To assess the effect of observer-model expertise differences on anticipation accuracy, observations for each model were analyzed by pooling the higher- and lower-skilled groups. The analysis of response rates for each clip among all participants identified significant differences between the models in some clips (Figure 2).

For kote, the unadjusted fisher’s exact test showed a significant difference in response rates between the high- and intermediate-level models in the second, sixth, and seventh clips (Figure 2a). In the second clip (up to −300 ms of video time), the percentage of “uncertain” responses was high, and the correct answer rate was zero for the intermediate-level model (χ^2^(2) = 6.234, unadjusted *p* = 0.035, *V* = 0.456). In the sixth and seventh clips (up to −120 ms and −90 ms, respectively), the correct answer rate for the intermediate-level model was higher than that for the high-level model (χ^2^(2) = 6.016, unadjusted *p* = 0.025, *V* = 0.455; χ^2^(1) = 5.00, unadjusted *p* = 0.046, *V* = 0.408, respectively).

For men, the unadjusted fisher’s exact test showed a significant difference in response rates between the high- and intermediate-level models in the first, fourth, and fifth clips (Figure 2c). In the first clip (up to −350 ms of video time), the percentage of “uncertain” responses was high for the intermediate-level model (χ^2^(2) = 6.528, unadjusted *p* = 0.038, *V* = 0.474). In the fourth and fifth clips (up to −200 ms and −150 ms, respectively), the correct answer rate was higher for the intermediate-level model than for the high-level model (χ^2^(2) = 6.716, unadjusted *p* = 0.035, *V* = 0.481; χ^2^ (2) = 6.429, unadjusted *p* = 0.040, *V* = 0.455, respectively).

**Figure 3:**
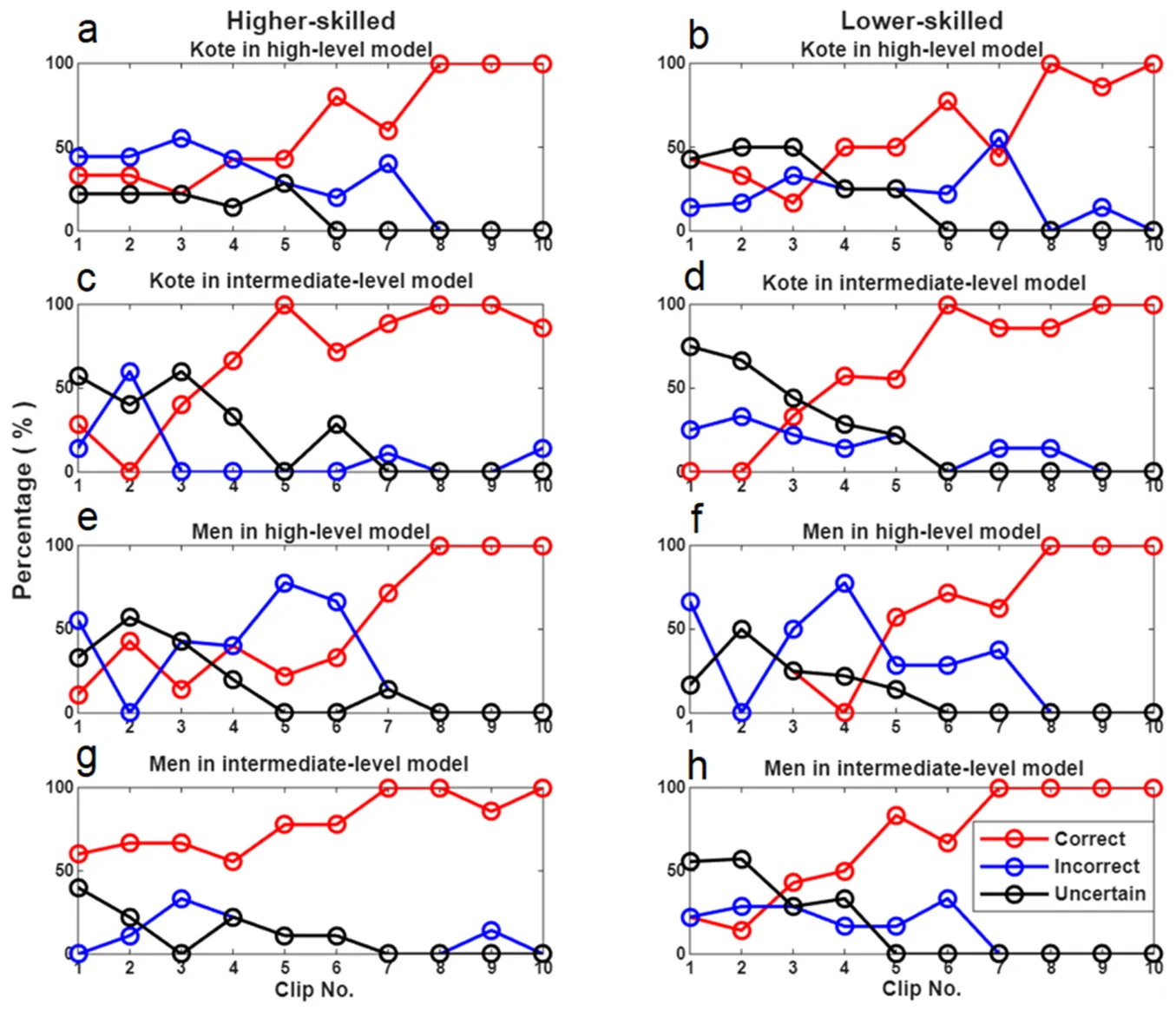
Response rates for both models’ kote and men clips according to group skill. (**a**–**d**): kote, (**e**–**h**): men.

Figure 3 shows the percentages of correct, incorrect, and “uncertain” responses for each clip per model, separately for kote and men, in the higher- and the lower-skilled groups. In both groups, the rate of correct answers increased toward the latter half of the clip, immediately before the strike. The chi-squared test revealed no significant difference in response rates between the groups (*p* > 0.05).

**Figure 4:**
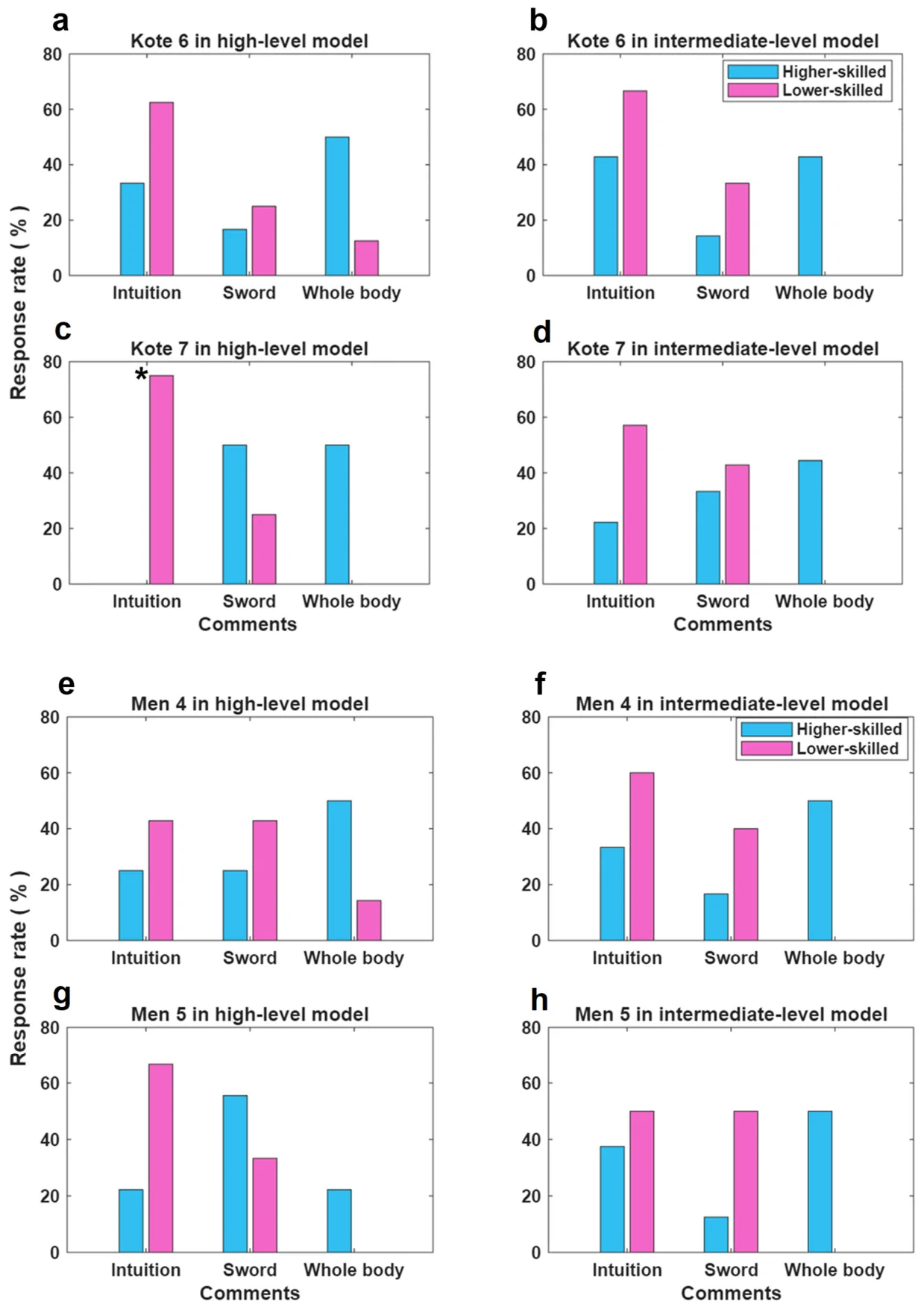
Response rates of comments in each skilled group. (**a**–**d**): kote in both models, (**e**–**h**): men in both models. Notes. *: Significant difference between the higher- and lower-skilled groups.

For the clips in which response rates significantly differed between the models (clips 6 and 7 for kote, excluding clip 2; clips 4 and 5 for men, excluding clip 1; the two excluded clips had a high percentage of “uncertain” responses), the percentages of reported reasons citing “intuition,” “sword tip,” and “whole body” as reasons were compared between the higher- and lower-skilled groups (Figure 4). Correct and incorrect answers did not differ significantly; therefore, both response types were analyzed. These four clips were selected for analysis because, in clips 1 and 2, participants relied exclusively on intuition when they did not provide an ‘uncertain’ response, whereas in the remaining clips, the orientation of the shinai provided an unambiguous cue regarding the striking direction.

For kote, the distribution of comments in the seventh clip of the high-level model differed between the higher- and lower-skilled groups (χ^2^(2) = 9.100, unadjusted *p* = 0.011, *V* = 0.806). In the lower-skilled group, the proportion of responses citing “intuition” was high; however, in the higher-skilled group, “intuition” was not cited, whereas the proportion of responses citing “whole body” was high (Figure 4c). No statistically significant differences in the distribution of reported reasons were found for the other clips (*p* > 0.05). For men, no significant difference was found in the percentage of comments between the higher- and the lower-skilled groups in any of the clips (*p* > 0.05, Figure 4e–h).

**Figure 5:**
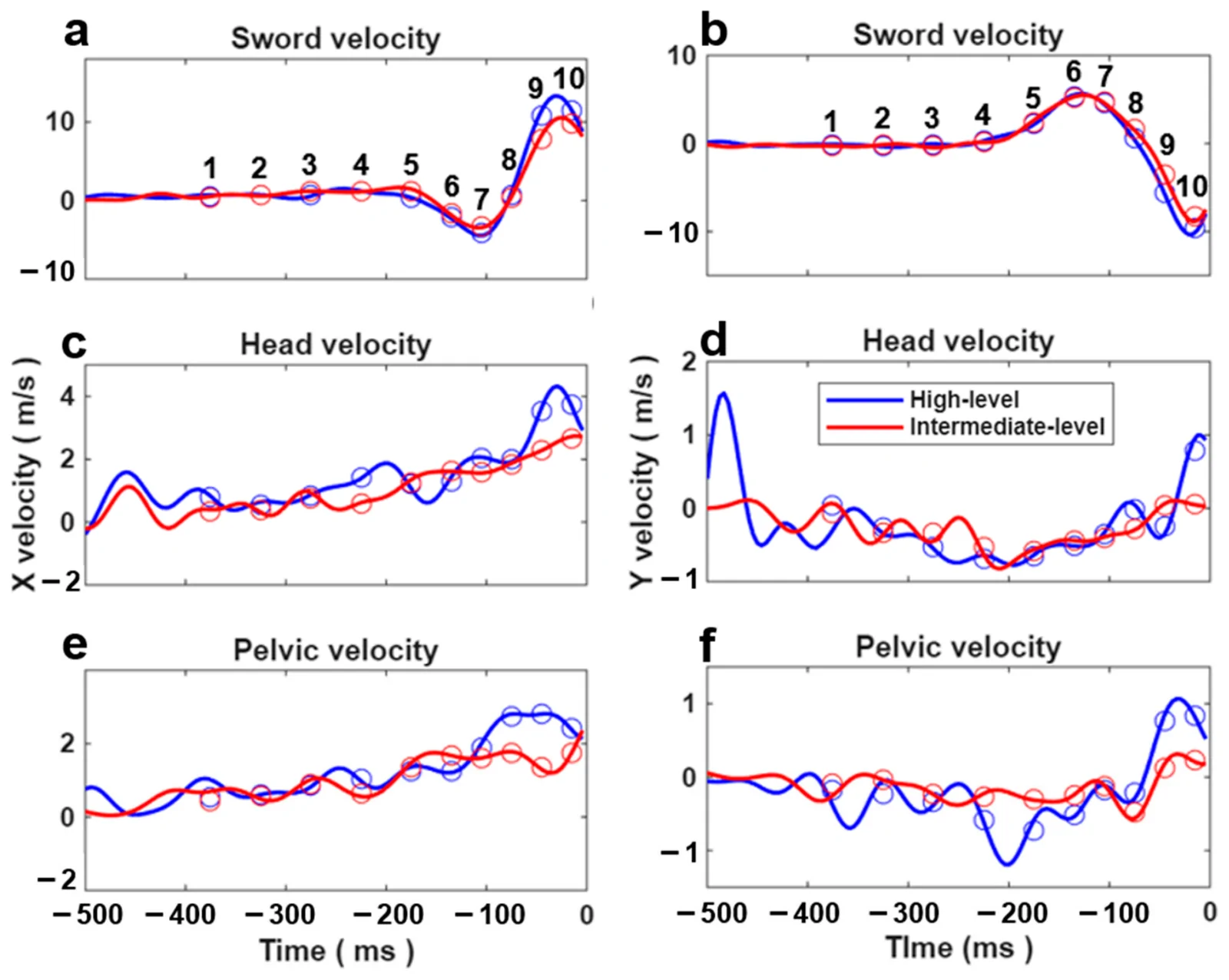
Velocity of each landmark in both models’ kote motion. (**a**,**b**): Sword in X and Y directions, respectively; (**c**,**d**): Head in X and Y directions, respectively; and (**e**,**f**): Pelvis in X and Y directions, respectively. Notes: The numbers on (**a**,**b**) show clip numbers.

**Figure 6:**
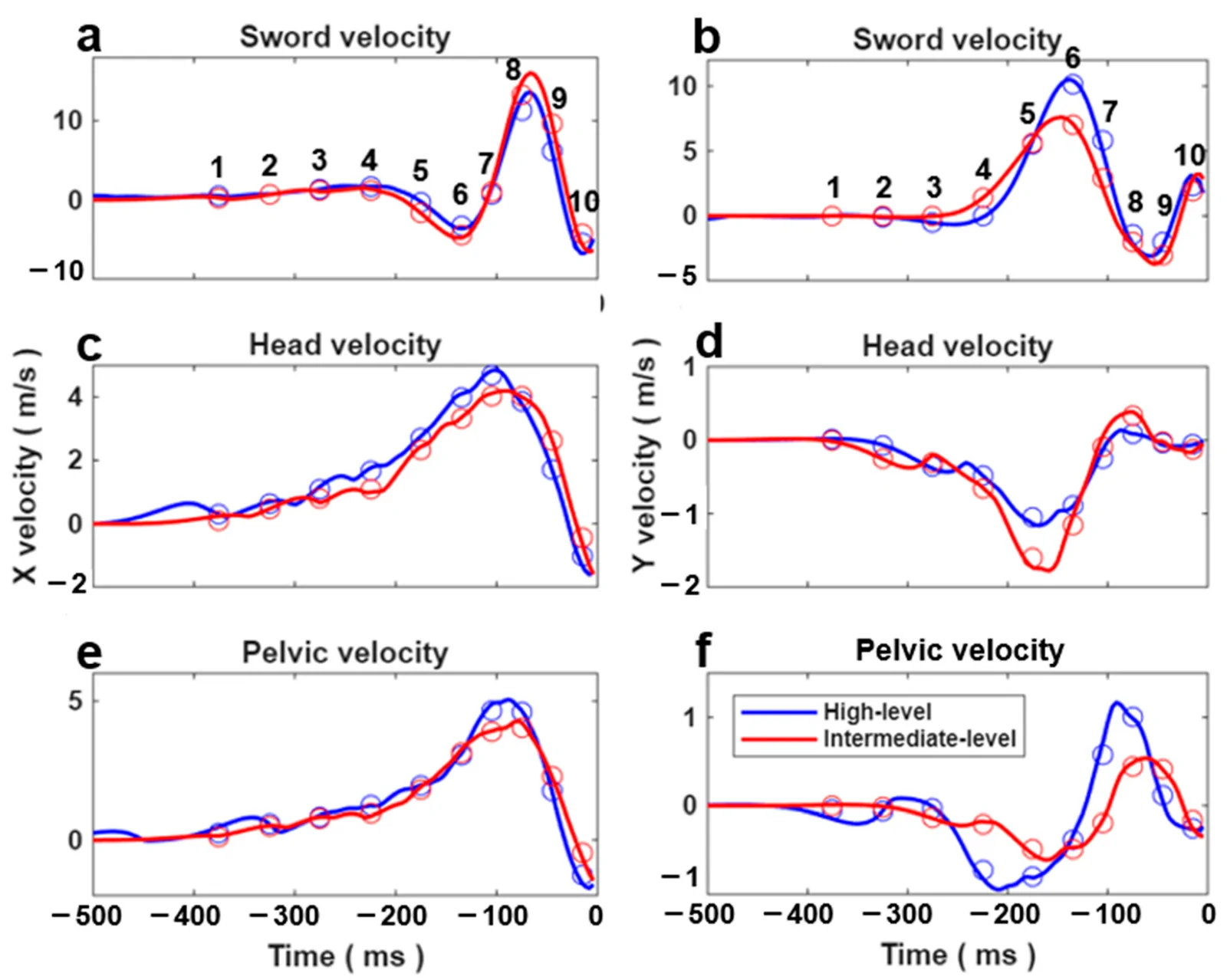
Velocity of each landmark in both models’ men motion. (**a**,**b**): Sword in X and Y directions, respectively; (**c**,**d**): Head in X and Y directions, respectively; and (**e**,**f**): Pelvis in X and Y directions, respectively. Notes: The numbers on (**a**,**b**) show clip numbers.

The movement patterns of the sword tip, head, and pelvis during kote and men were highly similar between the high- and intermediate-level models (Figure 5 and Figure 6, respectively, and Supplementary Materials). In the time period of the kote action clips in which response rates differed, the forward movement speed of the head and pelvis was slightly higher in the intermediate-level model (approximately 0.3 m/s) than in the high-level model during the sixth clip time frame (Figure 5c,e). In the seventh clip—the one in which the higher-skilled group frequently cited “whole body”—the forward movement speed of the pelvis in the high-level model was higher than that in the intermediate-level model and accelerated rapidly (Figure 5e). During the time period corresponding to the men action clips in which response rates differed, the upward speed of the sword tip and the downward speed of the head in the intermediate-level model were slightly higher (approximately 0.5 to 1.0 m/s) than those in the high-level model during the fourth clip time frame (Figure 6b,d).

## 4. Discussion

This study aimed to clarify whether knowledge of the opponent and competitive level, including the relative difference in expertise between observers and models, influence decision-making in kendo. Regarding knowledge of the opponent, a higher proportion of “uncertain” responses (accompanied by high effect sizes) was provided for the unfamiliar, intermediate-level model compared to the familiar, high-level model in stages of non-kinematic information (clips 1 and 2, Figure 2). Concerning the relative difference in expertise between observers and models, just before the strike, the correct answer rate for the intermediate-level model was significantly higher than that for the high-level model at the same timing (Figure 2). No statistically significant difference was found in the correct answer rate (i.e., prediction accuracy) between observers of different skill levels (Figure 3). However, regarding the cognitive process, the higher-skilled group used intuition less frequently than the lower-skilled group and utilized whole-body movement instead (Figure 4).

Regarding action anticipation accuracy (a core factor for rapid decision-making), influences from opponent knowledge and the relative difference in expertise between observers and models were observed in specific clips when analyzing the participants as a whole. In clip 2 for kote and clip 1 for men, there was a high proportion of “uncertain” responses with high effect sizes (Figure 2). If the difference in competitive level alone influenced decision-making, the intermediate-level model (with lower skill) should have elicited a lower rate of “uncertain” responses; however, the opposite was observed in these clips. Therefore, the fact that the “uncertain” rate was higher for the intermediate model suggests that knowledge of the opponent significantly influences decision-making. These clips correspond to the initial stages where almost no movement occurs, and kinematic cues are virtually absent (Figure 5 and Figure 6). Non-kinematic information (contextual priors) about an opponent plays a vital role in action anticipation and provides an advantage to experts in inferring intentions [8,10,11]. Thus, the low rate of “uncertain” responses for the high-level model likely stems from the availability of non-kinematic information (i.e., knowledge of the opponent’s habits or tendencies). While previous research has explored non-kinematic information [8,10,11], few studies have compared the presence or absence of familiarity with the opponent’s expertise level. We believe these findings hold significant value in this regard. Future research should examine non-kinematic cues in detail because opponent knowledge did not appear to influence decision-making in other clips lacking kinematic cues, such as clip 1 for kote or clip 2 for men.

Regarding the relative difference in expertise between observers and models, the correct answer rate was higher for the intermediate-level model in clips 6 and 7 for kote and clips 4 and 5 for men, showing high effect sizes (Figure 2). This suggests that observers can detect kinematic cues faster and more accurately when their own expertise level exceeds that of the model. According to the motion analysis results, the intermediate-level model showed a slightly faster increase in pelvis forward velocity (approx. 0.3 m/s) than the high-level model during the kote strike (Figure 5e). In the men strike, the intermediate-level model exhibited a larger downward displacement of the head compared to the high-level model (Figure 6d). Although these results are based on kinematic data from a single trial of one individual, we believe this is a highly suggestive finding, as to our knowledge, no prior studies have adjusted for the relative competitive level between observers and models. Future work could involve increasing the number of models to verify the validity of these findings and to determine whether they can be generalized within combat sports or extended to ball games.

Regarding the differences in action anticipation accuracy based on the observer’s competitive level, there was no statistically significant difference (Figure 3). Previous research has extensively compared anticipation between experts and novices [4,20,22] or among experts, novices, and coaches [1]. These studies have consistently shown that expert athletes generally possess a superior ability for action anticipation compared to novices across various ball sports [1,22] and combat sports [13,14,23]. However, findings from studies comparing elite athletes at different competitive levels vary depending on the specific action observed. In ball sports, it has been reported that in the presence of fakes (deceptive actions) in basketball, higher-level athletes may actually show lower accuracy than lower-level counterparts under certain constraints [20], while in handball, elite players demonstrate higher decision-making speed and accuracy than amateurs [24]. Regarding combat sports, while a systematic review suggests high-level athletes are generally more accurate than low-level ones [14], studies in fencing—a sport involving swords similar to kendo—found no significant difference in anticipation accuracy based on physical fitness levels among elite fencers [12]. Based on these reports and the findings of this study, it is possible that when the number of observed body parts and potential movement patterns increases, such as with the use of a “sword” or the presence of “fakes,” the difference in prediction accuracy between expert levels may become less pronounced. Future research is required to examine the impact on anticipation accuracy by varying the number of outcomes to be predicted within the same discipline.

While no difference was observed in overall anticipation accuracy between competitive levels, significant differences were found in the cognitive processes of decision-making for specific clips where correct answer rates varied between models (Figure 4c). Improving decision-making accuracy necessitates enhancing the precision of anticipation. Since anticipation is executed through motor simulation based on the observer’s own internal models [7], a broader motor repertoire within the internal movement representations leads to higher prediction accuracy [3,9]. To expand this repertoire, a process of error-based correction is required; therefore, the fact that higher-level participants had a lower proportion of “uncertain” or “intuitive” responses suggests that they engaged in more deliberate, model-based processing rather than mere guessing.

Furthermore, the focal point of their observation was not the distal tip of the bamboo sword but the whole-body kinematics. Kato [13] previously discovered using eye-tracking that experts employ a gaze anchoring strategy, which relates to the kendo-specific perceptual concept of “Enzan no Metsuke” (gazing at the far mountain) to capture the entire body. The findings of the present study, based on verbal reports of the participants’ actual cognitive focus, suggest that higher-level athletes may indeed utilize Enzan no Metsuke to perceive whole-body movements. As there have been no previous reports regarding the cognitive processes of decision-making in kendo, this finding provides an exploratory insight into the discipline’s unique internal mechanisms. In the future, it is necessary to clarify the perception-cognition mechanism by combining eye-tracking technology with verbal reports to further investigate the relationship between visual fixations and cognitive awareness.

This study has several limitations. First, regarding the sample size, although it was determined with reference to previous studies [1,17,18,19], it was found to be slightly insufficient based on a post hoc assessment. When the effect size was set to 0.45 or higher—a level that yielded significant results in the unadjusted tests within our statistical framework—a power analysis conducted via G*Power 3.1.9.7 (test family: *χ*^2^ tests; statistical test: goodness-of-fit tests: contingency tables; effect size *w* = 0.45; *α* err prob = 0.05; Power (1-*β* err prob) = 0.8; Df = 2) indicated that a total of 48 participants would be required to ensure adequate statistical power. As our current sample fell slightly short of this threshold, which may limit the generalizability of the findings, we have designated this work as “An exploratory study” in the title. Consequently, we intend to conduct further investigations with an expanded sample size in the future to verify the robustness of these preliminary results.

The second limitation concerns the statistical methodology. Because Fisher’s exact tests were conducted for each individual clip, we applied adjustments for multiple comparisons to mitigate the risk of Type I errors. Following these corrections, no statistically significant results were detected in any of the comparisons. As mentioned above, while we have characterized this as an exploratory study and focused our discussion on findings with a moderate effect size (Cramer’s *V* ≥ 0.45), these results remain largely speculative. The lack of significant differences after alpha-level correction means that the findings must be interpreted with caution. Therefore, further research is necessary to verify these trends by employing more robust statistical approaches and addressing the aforementioned sample size requirements to ensure adequate statistical power.

Third, the temporal segmentation of the observation videos was identical for both models. Consequently, the kinematic features of each model may have been captured at different stages. However, as shown in Figure 5 and Figure 6, the movement patterns of both models were highly similar, suggesting that temporal segmentation did not mask the characteristics of the models and that the observers were primarily observing the differences in movement between the models.

Fourth, although both groups included female observers, both action models were men. This likely did not affect the results, based on the finding of Meyer [20] that gender differences do not influence action anticipation, and because there was no difference in the male-to-female ratio between the observer groups in this study.

Fifth, using models with more distinct competitive levels has made it easier to detect differences related to the observers’ competitive levels. Additionally, differences might have been detected if the expert observers themselves were compared across a wide range of competitive levels. Future studies should also consider this perspective.

Sixth, this study utilized only two model players (one familiar and one novel) for the kinematic confirmation. While some previous studies have employed a similarly limited number of models for stimulus production (e.g., Denis et al. [9], who used two skilled coaches), this small sample size precludes the generalizability of the kinematic findings. Consequently, the kinematic information obtained from these models should be interpreted as supplementary data intended to provide exploratory insights into the observers’ cognitive processes rather than as a definitive representation of kendo expertise. To advance this line of inquiry into a more generalizable and robust study, it is necessary to recruit a larger number of models with comparable skill levels in future research.

The seventh limitation of this study was that a camera equipped with specialized lenses for distance correction was not utilized. This may have resulted in alterations to the participants’ field of view and perceptual-cognitive responses, potentially preventing the precise identification of the body regions or kinematic cues that observers naturally prioritize during a live match. Previous research in fencing has often relied on video footage recorded from a lateral (side-on) perspective [12]. While the present study’s use of a frontal perspective is a significant strength that enhances ecological validity by better replicating the viewpoint of an actual opponent in a competitive setting, it introduces technical challenges regarding spatial representation. Consequently, future research should employ recording equipment with focal lengths that more accurately accounts for the observer’s natural visual field and the realistic spatial dynamics between practitioners. To further refine our understanding of visual search strategies and action anticipation in kendo.

The final limitation is that the kinematic analysis of the models was conducted using only a single trial for each striking action. While the purpose of this analysis was to confirm the observation results, the possibility that these specific trials were anomalies (i.e., outliers) cannot be ruled out; therefore, the findings cannot be generalized. In future research, we plan to increase the number of models and trials per action to obtain more generalizable insights.

## 5. Conclusions

This study focused on decision-making in high school kendo athletes to clarify whether conditions integrating opponent knowledge and relative competitive level influence the cognitive process of decision-making. The athletes observed temporally occluded videos of strike actions performed by a familiar high-level model and an unfamiliar intermediate-level model. The findings suggest that knowledge of the opponent (serving as a contextual prior) supports decision-making at stages where kinematic information is absent. Furthermore, the relative difference in expertise between observers and models indicates an influence on cognitive processes during the period of decisional uncertainty immediately preceding the strike. In addition, higher-skilled athletes appeared to expand their internal motor repertoire by perceiving whole-body kinematics rather than relying on vague or intuitive perceptions of the opponent’s movements. By demonstrating these findings, this study provides new insights that advance the current decision-making paradigm in high-pressure sports contexts.

## Figures and Tables

**Table 1:** Demographic characteristics of the participants in both groups.

		Higher-Skilled Group		Lower-Skilled Group		Significance
		Mean	SD	Mean	SD	
Sex	Male	11		9		*1
	Female	4		6		
Age (years)		16.8	0.9	16.9	1.0	*2
Height (m)		168.7	8.2	165.8	7.0	*2
Weight (kg)		65.1	8.2	62.6	11.3	*2
Competitive experience (yrs)		11.3	2.1	9.5	3.2	*2
Grade	3rd Dan	12		11		*1
	2nd Dan	3		3		

*1: Differences are not statistically significant (*p* > 0.05, chi-squared test). *2: Differences are not statistically significant (*p* > 0.05, non-paired *t*-test).

**Table 2:** Duration of each action video clip.

Clip No	Start (ms)	End (ms)	Duration (ms)
1	−500	−350	150
2	−500	−300	200
3	−500	−250	250
4	−500	−200	300
5	−500	−150	350
6	−500	−120	380
7	−500	−90	410
8	−500	−60	440
9	−500	−30	470
10	−500	0	500

**Table 3:** Categories of reasons for the responses.

Category	Examples of Reported Reason
Intuition	Somehow
	Intuition
Sword tip	The tip of the sword rose or lowered
	The trajectory of the sword rose or dropped
	The left hand was raised or lowered
Body	The body moved down
	The body moved forward
	The body moved to the left

## Data Availability

The data presented in this study are available on request from the corresponding author due to participant privacy considerations and ethical restrictions imposed by the Institutional Re-view Board.

## Supplementary Materials

The following supporting information can be downloaded at: https://www.mdpi.com/article/10.3390/sports14080320/s1, Figure S1: Position and average velocity of each landmark in both models’ kote motion; Figure S2: osition and average velocity of each landmark in both models’ men motion.
