# Supplementary material for: Influence of Opponent Knowledge and Competitive Level on the Cognitive Processes of Decision-Making in High School Kendo Athletes: An Exploratory Study
Source: Sports (Basel). 2026 Aug 1;14(8):320. doi: 10.3390/sports14080320 (PMC13517098; doi:10.3390/sports14080320)
Supplement: Supplementary file 1 [file sports-14-00320-s001.zip › sports-4381448-supplementary.pdf]

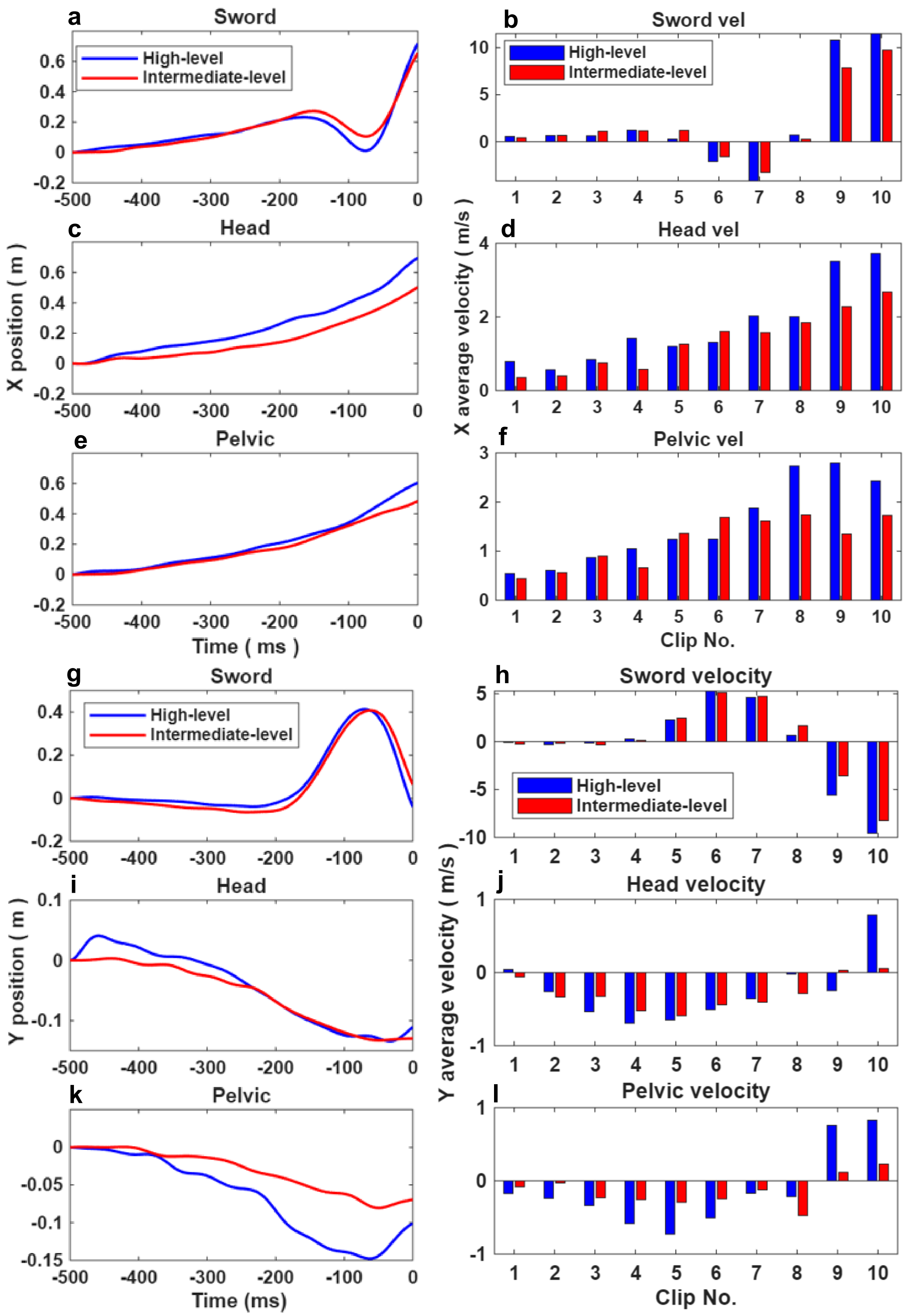

Supplementary Figure S1. Position and average velocity of each landmark in both models' kote motion. a–b and g–h: Sword in X and Y directions, respectively; c–d and i–j: Head in X and Y directions, respectively; and e–f and k–l: Pelvis in X and Y directions, respectively.

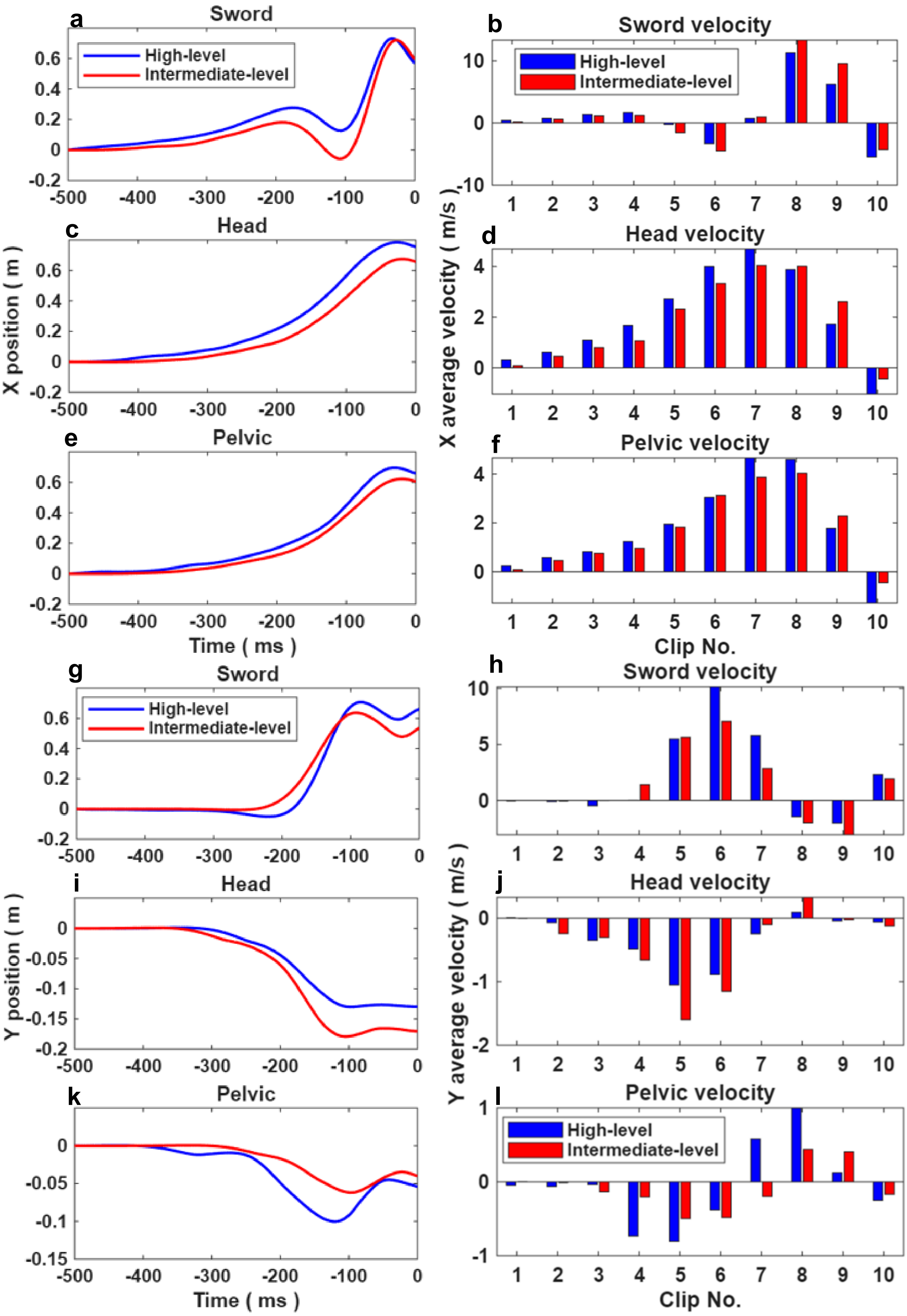

Supplementary Figure S2. Position and average velocity of each landmark in both models' men motion. a–b and g–h: Sword in X and Y directions, respectively; c–d and i–j: Head in X and Y directions, respectively; and e–f and k–l: Pelvis in X and Y directions, respectively.
